# miRNA expression profiling of 51 human breast cancer cell lines reveals subtype and driver mutation-specific miRNAs

**DOI:** 10.1186/bcr3415

**Published:** 2013-04-19

**Authors:** Muhammad Riaz, Marijn TM van Jaarsveld, Antoinette Hollestelle, Wendy JC Prager-van der Smissen, Anouk AJ Heine, Antonius WM Boersma, Jingjing Liu, Jean Helmijr, Bahar Ozturk, Marcel Smid, Erik A Wiemer, John A Foekens, John WM Martens

**Affiliations:** 1 Erasmus University Medical Center, Daniel den Hoed Cancer Center, Department of Medical Oncology and Cancer Genomics Center, Dr. Molewaterplein 50, 3015 GE Rotterdam, the Netherlands

## Abstract

**Introduction:**

Breast cancer is a genetically and phenotypically complex disease. To understand the role of miRNAs in this molecular complexity, we performed miRNA expression analysis in a cohort of molecularly well-characterized human breast cancer cell lines to identify miRNAs associated with the most common molecular subtypes and the most frequent genetic aberrations.

**Methods:**

Using a microarray carrying LNA™ modified oligonucleotide capture probes), expression levels of 725 human miRNAs were measured in 51 breast cancer cell lines. Differential miRNA expression was explored by unsupervised cluster analysis and was then associated with the molecular subtypes and genetic aberrations commonly present in breast cancer.

**Results:**

Unsupervised cluster analysis using the most variably expressed miRNAs divided the 51 breast cancer cell lines into a major and a minor cluster predominantly mirroring the luminal and basal intrinsic subdivision of breast cancer cell lines. One hundred and thirteen miRNAs were differentially expressed between these two main clusters. Forty miRNAs were differentially expressed between basal-like and normal-like/claudin-low cell lines. Within the luminal-group, 39 miRNAs were associated with *ERBB2 *overexpression and 24 with *E-cadherin *gene mutations, which are frequent in this subtype of breast cancer cell lines. In contrast, 31 miRNAs were associated with *E-cadherin *promoter hypermethylation, which, contrary to *E-cadherin *mutation, is exclusively observed in breast cancer cell lines that are not of luminal origin. Thirty miRNAs were associated with *p16^INK4 ^*status while only a few miRNAs were associated with *BRCA1, PIK3CA*/*PTEN *and *TP53 *mutation status. Twelve miRNAs were associated with DNA copy number variation of the respective locus.

**Conclusion:**

Luminal-basal and epithelial-mesenchymal associated miRNAs determine the subdivision of miRNA transcriptome of breast cancer cell lines. Specific sets of miRNAs were associated with *ERBB2 *overexpression, *p16^INK4a ^*or *E-cadherin *mutation or *E-cadherin *methylation status, which implies that these miRNAs may contribute to the driver role of these genetic aberrations. Additionally, miRNAs, which are located in a genomic region showing recurrent genetic aberrations, may themselves play a driver role in breast carcinogenesis or contribute to a driver gene in their vicinity. In short, our study provides detailed molecular miRNA portraits of breast cancer cell lines, which can be exploited for functional studies of clinically important miRNAs.

## Introduction

Numerous lines of evidence indicate that breast cancer is genetically and epigenetically not just one disease but a diverse group of diseases with diverse clinically relevant biological and phenotypical features. Recent technological advances in molecular profiling have led to the identification of an increasing number of molecular subtypes in breast cancer, each with distinct co-regulated and anti-regulated genes. However, the biology of these molecular subtypes and their underlying genetic drivers may be affected by numerous biological factors, including miRNAs.

miRNAs are a class of small nonprotein-coding genes that regulate the expression of genes post-transcriptionally via sequence-specific interaction with the 3' UTR of target mRNAs, resulting in inhibition of translation and/or mRNA degradation [1,2]. A large number of studies have established that miRNAs play essential roles in biological processes, such as development [3,4], cell proliferation [5], apoptosis [6], stress response, and tumorigenesis [7,8]. Aberrant expression levels of miRNAs have been observed in many solid cancers including breast cancer. In breast cancer, the expression levels of several miRNAs are significantly different between normal and cancerous tissues, between breast cancers of different molecular subtypes [9-11] with a different prognosis [12-14], and between breast cancers showing different responses to endocrine therapy [15,16]. Despite significant progress in the last few years on miRNA biology, the exact biological functions and the genetic factors driving their expression have been revealed for only a limited number of miRNAs in breast cancer.

Human breast cancer cell lines are excellent experimental models and renewable resources to investigate biological functions of clinically important miRNAs both in *in vitro *cultured conditions and *in vivo *when raised as xenografts [6,17-20]. Here, using microarrays we analyzed miRNA expression levels in 51 molecularly well-characterized human breast cancer cell lines. We explored the association of individual miRNA expression levels with intrinsic subtypes and the most common recurrent genetic aberrations. Through this analysis, we provide a catalog of miRNAs in human breast cancer cell lines, which can be used to understand underlying biology of clinically relevant miRNAs and to reveal the genetic factors that may be involved in their regulation.

## Materials and methods

### Breast cancer cell lines

The cohort of 51 human breast cancer cell lines used in this study is listed in Table 1. The origin of the cell lines has been described elsewhere [21]. Prior to expression profiling, all cell lines were established to be genetically unique, monoclonal and of correct identity by performing STR profiling using the PowerPlex^® ^16 system (Promega, Madison, WI, USA). The PowerPlex^® ^16 system included 15 STRs and one gender-discriminating locus (Penta E, D18S51, D21S11, TH01, D3S1358, FGA, TPOX, D8S1179, vWA, Amelogenin, Penta D, CSF1PO, D16S539, D7S820, D13S317, and D5S818).

**Table 1:** Molecular and biochemical characterization of 51 human breast cancer cell lines

Cell line	Molecular classification	Clinically relevant protein expression							
	
	**Intrinsic subtype**^**a**^	ER	PGR	ERBB2	EGFR	CK5	CK8-18	CK19	CK14
SUM185PE	Luminal	-	-	-	-	-	+	+	-
MDA-MB-175VII	Luminal	+	-	-	-	-	+	+	-
BT483	Luminal	+	-	-	-	-	+	+	-
T47D	Luminal	+	+	-	+	-	+	+	-
MDA-MB-415	Luminal	+	+	-	-	-	+	+	-
ZR75-1	Luminal	+	+	-	-	-	+	+	-
MCF-7	Luminal	+	+	-	-	-	+	ND	-
SUM52PE	Luminal	+	-	-	-	-	+	ND	-
UACC812	Luminal	+	+	++	-	-	+	+	-
MDA-MB-361	Luminal	+	+	++	-	-	+	+	-
ZR75-30	Luminal	+	-	++	-	-	+	+	-
SK-BR-5	Luminal	-	-	++	-	-	+	+	-
OCUB-F	Luminal	-	-	++	-	+	+	+	-
MPE600	Luminal	+	-	++	-	-	+	+	-
MDA-MB-134VI	Luminal	+	-	-	-	-	+	+	-
SUM44PE	Luminal	+	+	-	-	-	+	+	-
CAMA-1	Luminal	+	-	-	-	-	+	+	-
BT474	Luminal	+	+	++	-	-	+	+	-
MDA-MB-330	Luminal-ERBB2+	+	-	++	-	-	+	+	-
HCC1419	Luminal-ERBB2+	+	-	++	-	-	+	+	-
HCC202	Luminal-ERBB2+	-	-	++	-	-	+	+	-
SUM190PT	Luminal-ERBB2+	-	-	++	-	-	+	+	-
SUM225CWN	Luminal-ERBB2+	-	-	++	-	-	+	+	-
UACC893	Luminal-ERBB2+	-	-	++	+	-	+	+	-
SK-BR-3	Luminal-ERBB2+	-	-	++	-	-	+	+	-
EVSA-T	Luminal-ERBB2+	-	+	++	-	-	+	+	-
MDA-MB-453	Luminal-ERBB2+	-	-	++	-	-	+	+	-
HCC1569	ER-negative-ERBB2+	-	-	++	+	-	-	-	-
HCC1954	ER-negative-ERBB2+	-	-	++	+	+	+	-	-
HCC1500	ER-negative-ERBB2+	+	+	-	-	-	+	+	-
DU4475	Other	-	-	-	-	+	-	-	-
SUM229PE	Basal-like	-	-	-	+	+	+	+	-
HCC1937	Basal-like	-	-	-	+	+	+	-	-
MDA-MB-468	Basal-like	-	-	-	+	+	+	+	-
HCC1806	Basal-like	-	-	-	+	+	+	+	+
HCC70	Basal-like	-	-	-	+	+	+	+	-
HCC1143	Basal-like	-	-	-	+	+	+	+	-
BT20	Basal-like	-	-	-	+	+	+	+	-
SUM149PT	Basal-like	-	-	-	+	+	+	+	-
HCC1395	Basal-like	-	-	-	+	+	-	-	-
SK-BR-7	Normal-like/claudin-low	-	-	-	+	-	+	-	-
Hs578T	Normal-like/claudin-low	-	-	-	+	-	-	-	-
MDA-MB-231	Normal-like/claudin-low	-	-	-	+	-	-	-	-
SUM1315M02	Normal-like/claudin-low	-	-	-	+	-	-	-	-
MDA-MB-436	Normal-like/claudin-low	-	-	-	+	-	-	-	-
BT549	Normal-like/claudin-low	-	-	-	+	-	-	-	-
MDA-MB-157	Normal-like/claudin-low	-	-	-	+	-	-	-	-
SUM159PT	Normal-like/claudin-low	-	-	-	+	-	-	-	-
MDA-MB-435s	Normal-like/claudin-low	-	-	-	-	-	-	-	-
SUM102PT	Normal-like/claudin-low	ND	ND	ND	ND	ND	ND	ND	ND
HCC38	Normal-like/claudin-low	ND	ND	ND	ND	ND	ND	ND	-

CK, cytokeratin; ER, estrogen receptor; EGFR, epidermal growth factor receptor; PGR, progesterone receptor. ^a^Cell lines are classified based on expression of the intrinsic gene set defined by Perou and Colleagues [23]. For protein expression: +, expression; ++, overexpression; -, no expression; ND, not determined. Other subtype includes DU4475 since it is not classified to any subtype of breast cancer by the intrinsic gene set.

The method involved isolation of the genomic DNA from each breast cancer cell line using the QIAamp DNA Mini Kit (Qiagen, Hilden, Germany) and 10 ng of the isolated DNA was used as the input for the multiplex PCR. The following PCR conditions were used: 95°C for 11 minutes, 96°C for 1 minute, 10×(94°C for 30 seconds, 60°C for 30 seconds, 70°C for 45 seconds), 22×(90°C for 30 seconds, 60°C for 30 seconds, 70°C for 45 seconds), and then 60°C for 30 minutes. The PCR was carried out using primers linked with fluorescent dyes (6-carboxy-4',5'-dichloro-2',7'-dimethoxy-fluorescein, fluorescein, and carboxy-tetramethylrhodamine). The labeled amplicons were detected using the 3130*xl *Genetic Analyzer (Applied Biosystems, Foster City, CA, USA) and data were analyzed using the Genemarker 1.91 software from Softgenetics (State College, PA, USA).

The end result for each cell line was an electropherogram with each STR allele represented as one or more peaks of an appropriate fluorophore. The authenticity of all cell lines, except SUM cell lines, were assessed by comparing the generated STR profiles with the source STR profiles present in the American Type Culture Collection and the Deutsche Sammlung von Mikroorganismen und Zellkulturen. As no reference is available, the STR profiles of the SUM cell lines were matched with the profiles generated from the earliest passage of these cell lines stored in the in-house culture collection.

For experiments, each cell line was cultured in triplicate on collagen-coated petri dishes in RPMI 1640 medium supplemented with 10% heat-inactivated fetal bovine serum and antibiotic agents (100 μg/ml penicillin G and 80 μg/m streptomycin). The petri dishes were placed in a humidified atmosphere of 5% CO_2 _and 95% air at 37°C until cultures were 70 to 80% confluent. Ethical approval of our study was not necessary as our experiment involved only *in vitro *propagated human breast cancer cell lines.

### Total RNA isolation

Total RNA from all samples was isolated using RNAzol-B reagent (Campro Scientific BV, Veenendaal, the Netherlands) according to the manufacturer's manual. Briefly, a biological sample was lysed in RNAzol-B reagent and the lysate was separated into an aqueous phase and an organic phase after the addition of chloroform. DNA and protein were subsequently removed by carefully transferring the aqueous phase containing RNA to a fresh Eppendorf tube. The RNA was obtained from the aqueous phase by an isopropanol precipitation, washed with ethanol and air dried for subsequent procedures. The purity of the isolated RNA was checked using NanoDrop^® ^ND-1000 (Isogen Life Science, De Meern, the Netherlands) ensuring spectrophotometric ratios of A_260 nm_/A_280 nm _~2 and A_260 nm_/A_230 nm _≥ 2, and the quality control checks were performed according to the previously described methodology [22].

### Gene expression profiling

Total RNA (200 ng) was reverse transcribed, copied into double-strand cDNA and labeled to yield biotin-labeled cRNA using the 3' IVT express Kit (Affymetrix, Santa Clara, CA, USA) according to the manufacturer's instructions. Biotin-labeled cRNA was subsequently fragmented and loaded onto an Affymetrix GeneTitan instrument. The hybridization cocktail was applied to Human Genome HT_HG-U133_Plus_PM GeneChip 96-well arrays. All steps including hybridization, washing and scanning were carried out automatically inside the instrument. The raw data (.CEL files) were normalized by the RMA method using the default settings of the Affymetrix Expression Console^™ ^software and were used for statistical analysis. The microarray data were deposited in the Gene Expression Omnibus data repository [GEO:GSE41313].

For subtype classification of the cell lines, Perou and colleagues' intrinsic gene set of 496 genes [23] were matched to the Affymetrix probe sets using Unigene cluster numbers. Some of the genes have multiple probe sets present. To ensure analysis of only the informative probe sets, the probe sets that did not vary across all samples were removed, leaving the most variable ones for analysis (66% of the probe sets). These genes were then used to cluster the breast cancer cell lines. The intrinsic molecular subtypes were assigned as follows: luminal-type cell lines that exhibited higher expression of *ESR1, GATA3, TFF3*, and *FOXA1*; ERBB2-positive cell lines that showed higher expression of *ERBB2, GRB7*, and *STARD3*; basal-like cell lines, which were characterized by higher expression of *KRT5, KRT17, BST2*, and *FABP7*; and normal-like/claudin-low cell lines that did not show *KRT5 *and *KRT17 *expression and have low expression of claudin 3, claudin 4, and claudin 7 genes. The ERBB2-positive cell lines were further designated as luminal-ERBB2-positive and estrogen receptor (ER)-negative/ERBB2-positive cell lines, because the former in addition to *ERBB2 *overexpression also show *ESR1 *gene expression on microarray while the latter do not.

### miRNA expression analysis

miRNA expression profiling was performed using miRNA microarrays according to a previously published method [24]. In brief, 1 μg total RNA was labeled with Cy3 using the ULS aRNA labeling kit (Kreatech, Amsterdam, the Netherlands). The LNA™ modified oligonucleotide capture probe set (miRBase version 10.0, annotation version 13; Exiqon, Vedbaek, Denmark) was spotted in duplicate on Nexterion E glass slides in Nexterion Spot buffer (Schott, Elmsford, NY, USA) using a Virtek Chipwriter Pro (Bio-Rad, Hercules, CA, USA). The RNA sample with a labeling efficiency > 15 pmol Cy3/μg RNA was used for hybridization in a salt-based hybridization buffer (Ocimum Biosolutions, Hyderabad, India) overnight at 60°C using a Tecan HS4800 pro hybridization station. Hybridized slides were scanned in a Tecan LS Reloaded scanner. Data were extracted using Imagene software (6.0 standard edition; TCAN, Chapel Hill, NC, USA). The raw data were normalized using quantile normalization and used for statistical analysis. The normalized expression data are provided in Table S1 in Additional file 1. To validate our findings, subsets of the differentially expressed miRNAs between two miRNA-driven clusters were quantified using the Taqman Human MicroRNA Assay Set from Applied Biosystems (Nieuwerkerk aan den IJssel, the Netherlands) as described previously [12].

### SNP arrays

Genomic DNA from all cell lines was extracted using the QIAamp DNA Mini Kit (Qiagen). Genomic DNA (500 ng) was used as the starting material to capture genome-wide chromosomal aberrations with the aid of the Affymetrix Genome-Wide Human SNP 6.0 array technology. The steps were performed according to Affymetrix's recommended protocols. In summary, after digestion of the genomic DNA using either restriction enzymes NspI or StyI, adaptors were ligated to the obtained DNA fragments. These fragments were subsequently amplified using PCR, fragmented, end-labeled with biotin and hybridized onto GeneChip SNP 6.0 arrays. After hybridization the arrays were washed and scanned to generate the raw data (.CEL files) using the Affymetrix Genotyping Console™ software. The chromosomal gains and losses were calculated using SNP copy number variation (CNV) on the same chromosome. The chromosomal regions containing gains or losses were correlated with the expression level of the miRNAs located on the same genomic regions. The SNP data were deposited in the GEO data repository [GEO:GSE41313].

### Protein expression and mutational analysis

Protein expression data of ER, progesterone receptor, ERBB2, epidermal growth factor receptor, and cytokeratin (CK) 5, CK8-18, CK19, and CK14 were used from previously published work [25], except for 10 HCC cell lines that were characterized by immunohistochemistry using the same protocols as described before [25]. Mutational analysis of *p16^INK4a^, BRCA1, E-cadherin, PIK3CA*, and *PTEN *and promoter hypermethylation analysis of *E-cadherin *were previously reported for all cell lines except for HCC cell lines. These were separately analyzed according to the previously published methods [25] (data presented in Table S2 in Additional file 1).

### Hierarchical clustering and statistical analyses

Hierarchical clustering analyses of significant miRNAs were performed using Cluster 3 software [26] and the expression patterns of miRNAs and mRNAs in the heat maps were visualized using Treeview 1.1.6 R2. Average linkage clustering was carried out on both samples and mRNA and miRNA expression data, respectively, using Pearson correlation as a distance measure. Differential expression of miRNAs between two groups was determined using the univariate *t *test in BRB-array tools 3.7 developed by Dr. Richard Simon and the BRB-ArrayTools Development Team). A permutation value of *P *< 0.05 was considered statistically significant and used to select differentially expressed miRNAs for supervised hierarchical cluster analysis. The associations between continuous variables were tested using Spearman Rank correlation (*R*s values). The Kruskal-Wallis test was used to evaluate differences among groups and *P *< 0.05 was considered statistically significant.

## Results

### Molecular features of human breast cancer cell lines

The 51 human breast cancer cell lines that were used in this study are listed in Table 1, together with the protein expression results of ER, progesterone receptor, ERBB2, epidermal growth factor receptor and CK5, CK8-18, CK19, and CK14. All cell lines were first profiled for global mRNA expression using microarrays, and the cell lines in Table 1 are grouped into molecular subtypes based on the expression of the intrinsic genes originally described by Perou and colleagues [23].

**Figure 1:**
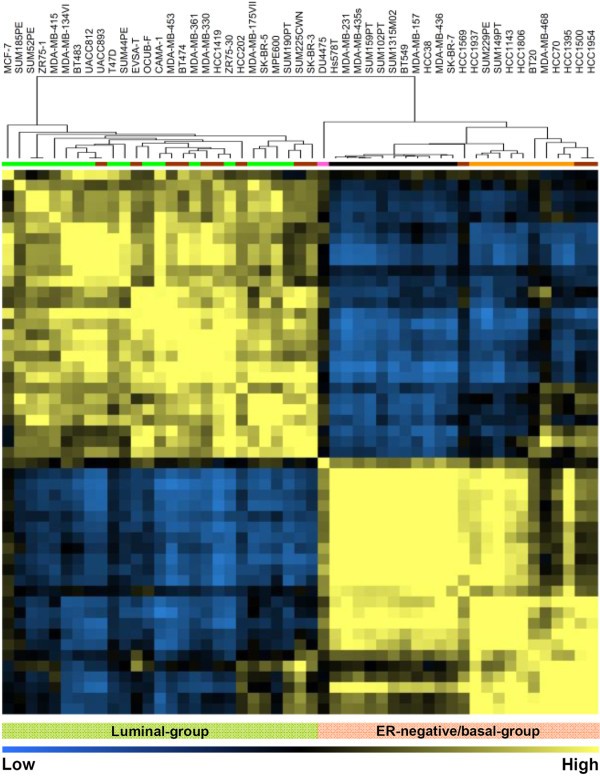
**Molecular subtyping of 51 human breast cancer cell lines**. Pearson correlation plot based on global mRNA expression of the top 10% variable genes. Breast cancer cell lines are depicted according to their overall similarity in gene expression. Yellow and blue, high and low overall similarity of samples in mRNA expression, respectively. Two main groups of 27 and 23 cell lines were apparent. Color codes for breast cancer subtypes based on intrinsic gene set: green, luminal-type cell lines; brown, luminal ERBB2-positive cell lines; black, normal-like/claudin-low cell lines; orange, basal-like cell lines; blue, estrogen receptor (ER)-negative/ERBB2-positive cell lines; pink, other subtype cell lines.

A Pearson correlation based on the top 10% of variably expressed genes classified the cell line cohort into two obvious groups (Figure 1). The first major group included 27 cell lines, of which 17 expressed ER protein. All cell lines in this group showed higher expression of luminal intrinsic genes as defined by Perou and colleagues [23]. We therefore defined this group of breast cancer cell lines as the luminal-group (Figure 1, left block). On the other hand, the second group included 23 breast cancer cell lines all of which were ER-negative and showed a predominant expression of basal intrinsic genes. We defined this group of breast cancer cell lines as "theER-negative/basal-group (Figure 1, right block). Moreover, according to classification using the intrinsic gene expressions [23], the luminal-group included nine ERBB2-overexpressing breast cancer cell lines, which did not cluster as a separate group. This result is in line with clustering of clinical specimens where the majority of luminal, ERBB2-positive tumors cluster with the luminal B tumors [27]. Within the ER-negative/basal-group, the basal-like and normal-like/claudin-low cell lines clustered distinctively into two subgroups (Figure 1). The DU4475 cell line could not be assigned to any subtype using the intrinsic gene set and was therefore designated as "the other" subtype.

Notably, the entire luminal-group of 27 cell lines expressed luminal cytokeratins (such as CK8/18 and CK19). The basal-like cell lines within the ER-negative/basal-group expressed basal and luminal cytokeratins (CK5, CK19, CK8-18), whereas all normal-like/claudin-low cell lines lacked the expression of these cytokeratins except for SK-BR-7 that exhibited CK8-18 expression (Table 1). Furthermore, except for MDA-MB-435s, the entire ER-negative/basal-group of 23 cell lines also expressed epidermal growth factor receptor protein (Table 1), which is a known marker for basal-type breast cancer.

### miRNA expression profiling revealed two groups of breast cancer cell lines

**Figure 2:**
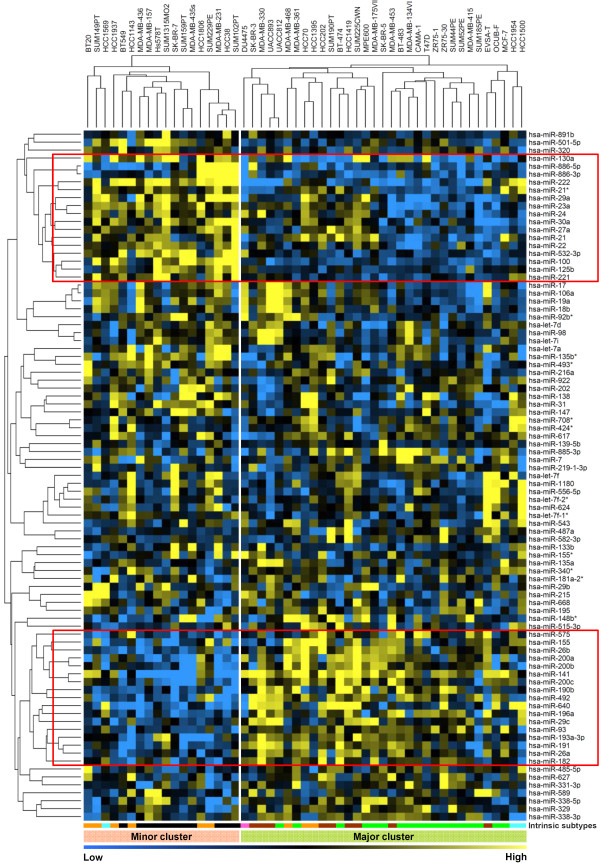
**Global miRNA expression analysis of 51 human breast cancer cell lines**. Unsupervised clustering across the breast cancer cell lines using the 87 most variably expressed miRNAs. Yellow and blue, high expression and low expression of the particular miRNA in the particular sample, respectively. Cell lines in the dendrogram of the hierarchical clustering based on miRNA expression are coded according to their intrinsic subtypes and depicted at the bottom of the figure (for color codes see Figure 1 caption). Red boxes enlist the groups of miRNAs that show the most robust cluster-specific differential expression in the cell lines. miRNA *hsa-miR-21 *in the red box of minor cluster failed to reach our criteria of fold change ≥ 1.5.

miRNA expression profiling was carried out using microarrays containing LNA™ capture probe sets against 725 human miRNAs. After filtering out miRNAs that were undetectable in most of the cell lines (57%), 413 miRNAs remained and were selected for further analysis. Unsupervised hierarchical clustering based on the most variable 87 miRNAs (with standard deviation ≥ 0.75) revealed two prominent cell line clusters: a minor cluster that included 18 cell lines and a major cluster that included 33 cell lines (Figure 2). Based on intrinsic subtyping, 17 of 18 cell lines present in the minor cluster belonged to either the basal-like or the normal-like/claudin-low subtypes, and interestingly 16 of them were deficient of ER, progesterone receptor and ERBB2 protein expression (triple-negative). The other two cell lines (HCC38 and SUN102PT) remained undetermined for protein analysis due to technical reasons. On the other hand, 29 of 33 breast cancer cell lines present in the major cluster were of the luminal-type, and all cell lines except SUM185 exhibited either one of the hormone receptor proteins or showed ERBB2 protein expression. The DU4475 cell line, which was previously proposed to have a stem cell-like phenotype due to activation of the Wnt signaling pathway [25,28], clustered at the edge of the major cluster. As a whole, similar to mRNA expression, miRNA expression profiling discriminated cell lines into two major subtypes of breast cancer.

Among the 87 most-variable expressed miRNAs across the entire panel, a group of 15 miRNAs (hsa-miR-130a, hsa-miR-886-5p, hsa-miR-886-3p, hsa-miR-222, hsa-miR-21*, hsa-miR-29a, hsa-miR-23a, hsa-miR-24, hsa-miR-30a, hsa-miR-27a, hsa-miR-22, hsa-miR-532-3p, hsa-miR-100, hsa-miR-125b, hsa-miR-221) was significantly higher expressed in the minor cluster as opposed to other miRNAs (Figure 2, top red box). Another group of 17 miRNAs (*hsa-miR-575, hsa-miR-155, hsa-miR-26b, hsa-miR-200a, hsa-miR-200b, hsa-miR-141, hsa-miR-200c, hsa-miR-190b, hsa-miR-492, hsa-miR-640, hsa-miR-196a, hsa-miR-29c, hsa-miR-93, hsa-miR-193a-3p, hsa-miR-191, hsa-miR-26a, hsa-miR-182*) showed significantly higher expression in the major cluster compared with the other miRNAs (fold change ≥ 1.5) (Figure 2, bottom red box).

To confirm the validity of miRNA array data, we had previously measured miRNA expression levels by real-time RT-PCR in our laboratory available from 20 miRNAs differentially expressed between the major cluster and the minor cluster for four ER-positive cell lines and four ER-negative cell lines. On average, the expression levels of these 20 miRNAs in these eight cell lines measured on two different platforms showed a positive correlation (*R*s = 0.53 and *R*s = 0.35 for miRNAs that were positively and negatively associated with ER status, respectively) (see Table S3 in Additional file 1). These data indicate that the differential expression of these miRNAs measured by the LNA™-based microarray in most of the cases could be validated by an independent technique.

As the miRNA-driven major and minor clusters included ER-positive (18 of 33) and ER-negative (all) cell lines, respectively, we determined whether differential expression of miRNAs between the two clusters is driven by ER status of the cell lines. For this analysis, we grouped cell lines into ER-positive and ER-negative based on mRNA expression of ER and performed supervised cluster analysis on these groups. We identified 79 differentially expressed miRNAs between ER-positive and ER-negative cell lines (*P *< 0.05) (see Figure S1 in Additional file 2 and Table S4A in Additional file 1), of which 54 miRNAs were common to the 113 miRNAs that were differentially expressed between the two clusters identified through unsupervised analysis (see Table S5 in Additional file 1). Interestingly, however, 59 miRNAs were uniquely differentially expressed between the two miRNA-driven clusters whereas 25 miRNAs were uniquely related to ER status of the cell lines (see Table S6 in Additional file 1). To reveal whether ER-related miRNAs in cell lines could be associated with the ER status in clinical tumors, we used a publicly available dataset from Cimino and colleagues comprising 77 primary breast tumors (53 ER-positive tumors and 24 ER-negative tumors) [29] and determined differentially expressed miRNAs between ER-positive and ER-negative tumors. Among 37 miRNAs commonly detectable in our data and in Cimino and colleagues' dataset, the differential expression of six miRNAs showed an overlap between cell lines and primary tumors. Notably, however, five of these six miRNAs (*hsa-miR-141, hsa-miRNA-26a, hsa-miR-29c, hsa-miR-148b, hsa-miR-193a-3p*) showed significantly higher expression in both ER-positive cell lines and primary tumors, and one miRNA (*hsa-miR-532-3p*) showed significantly lower expression in both ER-positive cell lines and ER-positive tumors (see Table S4B in Additional file 1).

### miRNA expression of cell lines associated with molecular intrinsic subtypes of breast cancer

**Figure 3:**
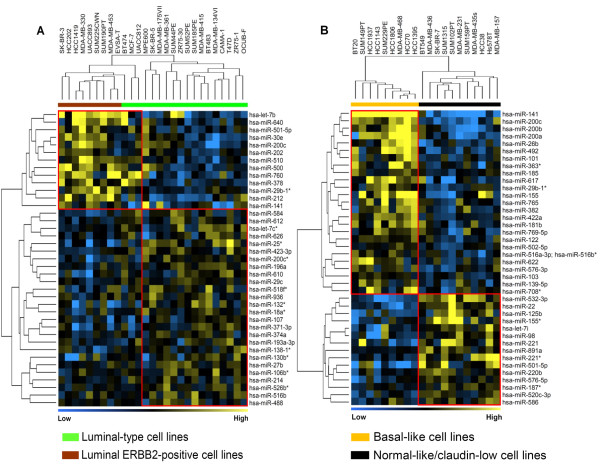
**Molecular subtype-specific differential expression of miRNAs**. Differentially expressed miRNAs **(A) **between luminal-type and luminal ERBB2-positive breast cancer cell lines within the mRNA-derived luminal-group (see Figure 1), and **(B) **between basal-like and normal-like/claudin-low breast cancer cell lines within the mRNA-derived estrogen receptor-negative/basal-group. Yellow and blue, high and low overall similarity of samples in miRNA expression, respectively.

We next aimed to identify miRNAs that are differentially expressed between the intrinsic subtypes of breast cancer. As expected, we observed a substantial overlap of differentially expressed miRNAs between ER-positive and ER-negative cell lines and the luminal-group and ER-negative/basal-group of cell lines (see Figure S1 in Additional file 2 and Tables S4A and S7 in Additional file 1). To identify additional miRNAs that are related to intrinsic subtypes but to avoid the confounding effect of ER, we compared cell lines with and without ERBB2 overexpression within the luminal-group. Likewise we compared the intrinsic basal-like cell lines with normal-like/claudin-low cell lines within the ER-negative/basal-group. These comparisons revealed 39 differentially expressed miRNAs in luminal cell lines with or without overexpressed ERBB2; 40 miRNAs were found differentially expressed between basal-like and normal-like/claudin-low cell lines (*P *< 0.05) (Figure 3A, B; see Tables S8 and S9 in Additional file 1).

The top most highly expressed miRNAs in ERBB2 overexpressing cell lines included *hsa-let-7b, hsa-miR-640, hsa-miR-200c, hsa-miR-378, hsa-miR-141, hsa-miR-196a, hsa-miR-29c*, and hsa-miR-18a*, whereas *hsa-miR-501-5p, hsa-miR-202, hsa-miR-760*, and *hsa-miR-626 *were more highly expressed in luminal cell lines lacking ERBB2 overexpression (fold change ≥ 1.5) (see Table S8 in Additional file 1). Similarly, the most highly expressed miRNAs in normal-like/claudin-low cell lines were *hsa-miR-22, hsa-miR-532-3p, hsa-miR-125b, hsa-miR-501-5p*, and hsa-miR-155*, whereas in basal-like cell lines miRNAs of the miR-200 family (*hsa-miR-492, hsa-miR-26b, hsa-miR-617, hsa-miR-155*) were highly expressed (fold change ≥ 2) (see Table S9 in Additional file 1). Notably, hsa-miR-155* and *hsa-miR-155*, which derived from the same precursor, showed an opposing expression pattern in basal-like compared with normal-like/claudin-low breast cancer cell lines.

### Differential expression of miRNAs and *E-cadherin *loss in breast cancer cell lines

**Figure 4:**
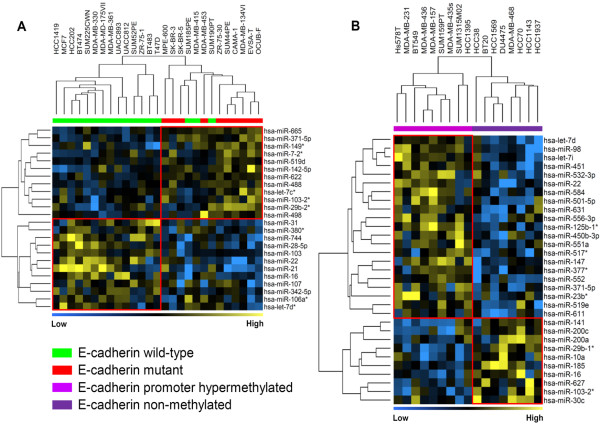
**Differential expression of miRNAs with respect to *E-cadherin *status**. Differentially expressed miRNAs **(A) **between *E-cadherin *mutant and wild-type breast cancer cell lines in the luminal-group, and **(B) **between *E-cadherin *promoter hypermethylated and wild-type breast cancer cell lines in the estrogen receptor-negative/basal group. Cell lines HCC1806, SK-BR-7, SUM102PT, SUM149PT, and SUM229PE, which show partial promoter hypermethylation, were not included in the analysis since we were not sure to which extent partial promoter methylation will affect *E-cadherin *expression levels in these cell lines. Yellow and blue, high and low overall similarity of samples in miRNA expression, respectively.

E-cadherin elicits a growth suppressive effect in mammary epithelial cells and its expression is frequently lost in human breast tumors [30]. Loss of E-cadherin expression occurs either by gene mutation or by gene promoter hypermethylation. In this cohort of cell lines and in clinical specimens, *E-cadherin *is preferentially mutated in luminal/ER-positive cell lines while it is frequently inactivated by promoter hypermethylation in basal/ER-negative cell lines [21]. To determine whether loss of *E-cadherin *expression in human breast cancer cell lines is associated with differential expression of miRNAs, we compared 10 *E-cadherin *mutant cell lines with 17 wild-type cell lines within the luminal-group of cell lines (Figure 4A) and compared nine *E-cadherin *promoter hypermethylated cell lines with eight nonmethylated cell lines within the ER-negative/basal-group (Figure 4B). Our comparison revealed signatures of 24 and 31 differentially expressed miRNAs associated with *E-cadherin *mutant and wild type cell lines and with cell lines having *E-cadherin *promoter hypermethylation or not, respectively (*P *< 0.05) (Figure 4A, B; in see Tables S10 and S11 in Additional file 1). Interestingly, both E-cadherin signatures had no miRNAs in common showing similar behavior.

### Association of miRNAs expression with common genetic aberrations (*p16^INK4a^, TP53, BRCA1, PIK3CA, PTEN*)

The *BRCA1, p16^INK4a^, TP53*, and *PTEN *genes play a growth suppressive role in mammary epithelial cells and are frequently inactivated in human breast tumor tissue. *PIK3CA *is also frequently mutated in breast cancer but has an oncogenic role. To reveal differentially expressed miRNAs associated with these mutations, we performed supervised analyses between mutant and wild-type cell lines for these genes. To avoid the confounding effects of ER, we performed these analyses only in ER-positive or ER-negative cell lines.

**Figure 5:**
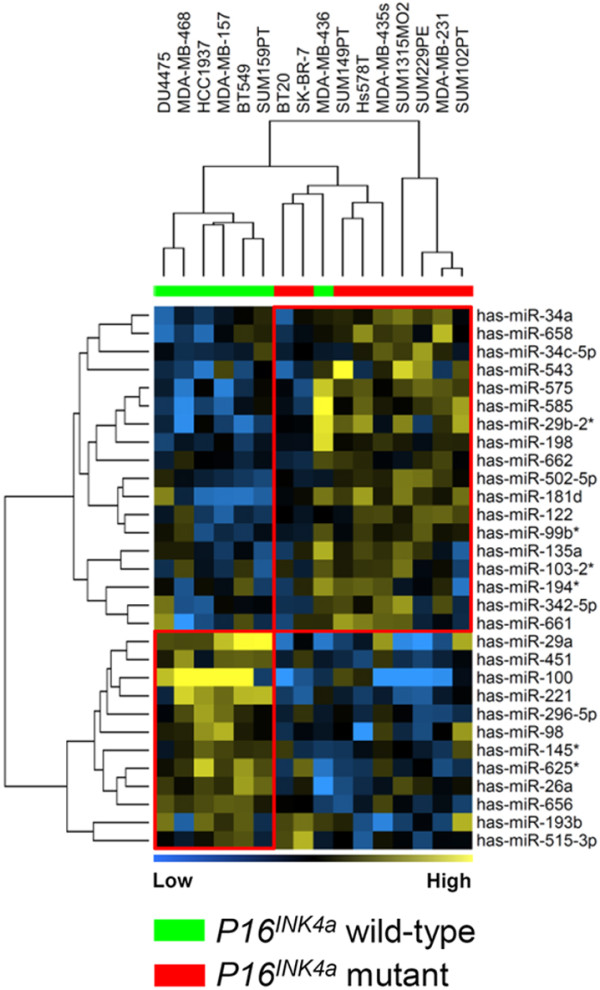
**Differentially expressed miRNAs between *p16^INK4a ^*mutant and wild-type cell lines in the estrogen receptor-negative/basal-group**. The analysis was restricted to only the estrogen receptor (ER)-negative/basal-group of cell lines since the majority of *p16^INK4a ^*mutant cell lines were ER-negative. Yellow and blue, high and low overall similarity of samples in miRNA expression, respectively.

In our cohort, 13 cell lines had lost the *p16^INK4a ^*locus, of which the majority are ER-negative. The comparison revealed that 30 miRNAs were differentially expressed between nine p16^INK4a ^mutant cell lines and seven wild-type cell lines in the ER-negative/basal-group (Figure 5; see Table S12 in Additional file 1). The most highly expressed miRNAs in *p16^INK4a ^*mutant cell lines were *hsa-miR-29a *and *hsa-miR-100 *(fold change ≥ 2). Notably, the *p16^INK4a ^*miRNA signature in the ER-negative/basal-group of cell lines appeared to be pronounced, and when used for clustering could almost completely separate the cell lines mutant for the *p16^INK4a^*gene from those that were wild-type. One should, however, emphasize that the tumor suppressor genes *p16^INK4a ^*and *p14^ARF ^*are co-localized and are often simultaneously lost amongst cell lines. We therefore cannot determine which of these genes might drive the differential expression of these miRNAs.

Furthermore, the current cohort of breast cancer cell lines contained 15 *PIK3CA *mutant cell lines (four ER-negative, eleven ER-positive) and eight *PTEN *mutant cell lines (four ER-negative, four ER-positive). Both *PIK3CA *and *PTEN *are components of the PI3K signaling pathway and mutated mutually exclusively in clinical specimens [31] and also in this cohort of cell lines [32]. Separate analyses between *PIK3CA *or *PTEN *mutant versus wild-type cell lines for these genes did not yield a considerable number of differentially expressed miRNAs. We therefore combined all cell lines harboring mutations either in *PIK3CA *or *PTEN *and compared them with wild-type cell lines. As *PIK3CA/PTEN *mutations are present in both the luminal-group and the ER-negative/basal-group of cell lines, we therefore performed multiple analyses. First, we analyzed all cell lines and found 49 miRNAs, which were differentially expressed between mutant and wild-type cell lines (see Figure S2A in Additional file 2 and Table S13 in Additional file 1). With successive analyses, we found eight and 42 differentially expressed miRNAs among the ER-negative/basal-group and the luminal group, respectively (see Figure S2A in Additional file 2 and Tables S14 and S15 in Additional file 1).

With respect to *BRCA1*, which is mutated in four of the ER-negative cell lines, we identified 13 miRNAs being differentially expressed between four mutant cell lines and 12 wild-type breast cancer cell lines (see Figure S2B in Additional file 2 and Table S16in Additional file 1). The two most highly expressed miRNAs in *BRCA1 *mutant cell lines were *hsa-miR-29b *and *hsa-miR-891b *(fold change ≥ 2). Finally, we analyzed differentially expressed miRNAs between *TP53 *mutant cell lines (*n *= 39) and wild-type cell lines (*n *= 9) and found 18 to be associated with the *TP53 *mutation status (see Figure S3 in Additional file 2 and Table S17 in Additional file 1). Important to note, however, is that the differentially expressed miRNA signatures associated with *BRCA1, PIK3CA/PTEN*, and *TP53 *mutations did not strongly discriminate between mutant and wild-type cell lines, and therefore their significance may be of limited value.

### miRNA genes show genomic aberrations in breast cancer cell lines

**Figure 6:**
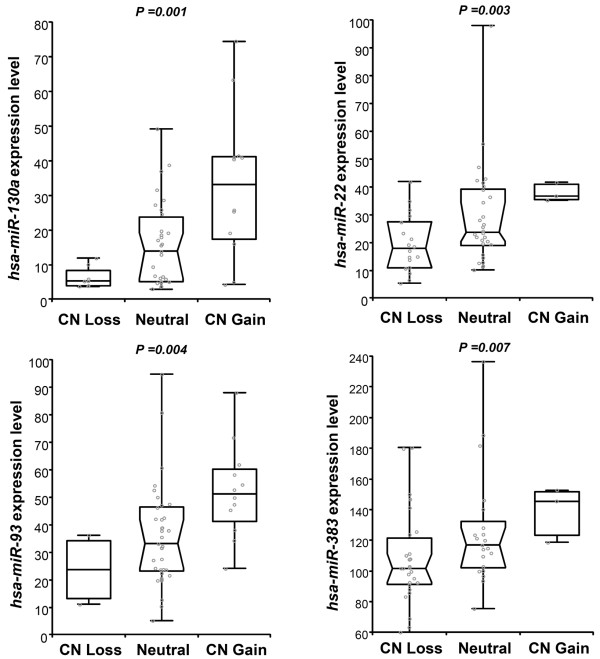
**Association of miRNA expression with genomic copy number variation in breast cancer cell lines**. The top four most significant miRNAs are represented (see Table S16 in Additional file 1 for a complete list). The Kruskal-Wallis test was used to reveal significant associations of miRNAs with genome copy number (CN) variation. *y *axis, expression levels of miRNA; *x *axis, number of samples with CN loss/gain or neutral.

Previous studies show that DNA CNVs in breast tumor tissues can lead to differential expression of genes and miRNAs [33-35]. To investigate this in our cohort of breast cancer cell lines, we first determined the DNA CNVs of the cell lines by performing whole-genome SNP profiling. These CNVs were then correlated with the expression levels of the 87 most variably expressed miRNAs in the cell lines. The correlation revealed 12 miRNAs, which were significantly associated with DNA CNVs (Kruskal-Wallis test, *P *< 0.05) (see Table S18 in Additional file 1). The top four most significantly associated miRNAs - *hsa-miR-130a *(11q12.1), *hsa-miR-22 *(17p13.1), *hsa-miR-93 *(7q22.1) and *hsa-miR-383 *(8p22) - with DNA CNVs in breast cancer cell lines are shown in Figure 6.

## Discussion

Human breast cancer cell lines are renewable resources that are extensively utilized as reliable workhorses to explore biological functions of clinically relevant molecules in breast cancer. Extensive molecular characterization and gene mutation analysis by us and other researchers have suggested that breast cancer cell lines have retained significant molecular features that are commonly observed in clinical breast tumors [36,37]. This suggestion prompted us to use 51 human breast cancer cell lines as a discovery cohort to identify differentially expressed miRNAs between known intrinsic subtypes of breast cancer as well as those associated with common genetic aberrations present in the cell lines.

In this study, we demonstrated that global miRNA expression profiling can assign our cell line collection into two clusters. These two clusters predominantly mirror the ER-based dichotomy present in human breast cancer cell lines, which may point to the fact that, like mRNA, miRNA expression profiling allows the discrimination between ER-positive and ER-negative cell lines. This suggests that a significant number of miRNAs may be under the control of ER regulation. In line with this, we observed a trend of finding ER binding sites closer to the significantly differentially expressed miRNAs between ER-positive and ER-negative cell lines than nonsignificant miRNAs (see Table S19 in Additional file 1 and Figure S4 in Additional file 2). Interestingly, not all miRNAs discriminating ER-positive and ER-negative cell lines are associated with ER status and therefore the division of the cell lines into two clusters may not be purely dictated by the expression of ER-related miRNAs, but these miRNAs are probably also related to luminal versus basal cellular differentiation.

Our supervised analysis revealed a signature of 79 differentially expressed miRNAs between ER-positive and ER-negative cell lines. Six of these miRNAs were also found similarly differentially expressed between ER-positive and ER-negative primary tumor specimens as they were in cell lines [29] (see Table S4B in Additional file 1). This overlap may seem small but we have observed discrepancies between miRNA profiling platforms (unpublished observations), and our study used LNA™ technology-based microarrays to quantify miRNA expression in the cell lines whereas Cimino and colleagues used Agilent microarrays to measure miRNAs in tumors. Also not all miRNAs are present on both platforms, which already explains one-half of the discrepancies. Furthermore, tumor heterogeneity and stromal contribution (such as connective tissues, blood vessels and immune infiltrates) and a likely selection bias in the cell lines - which are more ER-negative, mesenchymal, and *TP53 *mutant than primary tumors - could explain the observed discrepancies. A perfect correlation would thus not have been anticipated. Reassuringly, however, we found miRNAs previously reported to be ER-regulated miRNAs, such as a cluster of *hsa-miR-221/222 *[12,38]. In line with this, we also found a significant inverse correlation between the expression levels of these ER-regulated miRNAs and mRNA expression levels of their functionally validated target genes - for instance, *hsa-miR-221*and *hsa-miR-222 *have been shown to target the *CDKN1B *and *ESR1*genes (see Table S20 in Additional file 1). Such miRNAs have been shown to interact directly with ER and cause a phenotypic shift from ER-positive to ER-negative tumor cells [38]. Our miRNA analyses in this cohort of cell lines therefore confirms previous findings but also revealed new miRNAs (discussed below), which may have potentially interesting biological roles in ER-driven cancer.

Among these potentially novel ER-related miRNAs, four miRNAs (*hsa-miR-26a, hsa-miR-92b, hsa-miR-191, hsa-miR-492*) appear to show consistently higher expression across all the ER-positive cell lines (fold change ≥ 1.5), and as yet only *hsa-miR-26a *has been implicated in breast carcinogenesis whereas *hsa-miR-26a *and *hsa-miR-92b *have also been implicated in brain tumors [39,40]. In breast cancer, high expression of the *hsa-miR-26a *miRNA downregulates *EZH2 *and is therefore related to a favorable outcome on tamoxifen in metastatic breast cancer [41], and also interacts with *CDK4 *and *CENTG1 *oncogenes and forms an integrated oncomir/oncogene DNA cluster, which promotes glioblastoma tumor growth via RB1, PI3K/AKT, and JNK pathways [39]. On the other hand, *hsa-miR-92b *has been found to be exclusively overexpressed in primary brain tumors but serves as a biomarker to discriminate brain primary cancer from metastasis [40]. The other two miRNAs (*hsa-miR-191, hsa-miR-492*) have been linked to hepatic cancer. The increased expression of *hsa-miR-191 *stimulates proliferation in hepatocellular carcinoma cell lines and its therapeutic targeting suppresses tumor masses *in vivo *[42]. The *hsa-miR-492 *miRNA has been shown to be processed from the *keratin 19 *gene and upregulated in metastatic hepatoblastoma [43]. How these miRNAs play their biological roles in the context of breast cancer remains unknown and demands clinical and functional validation studies.

Another major contribution to the overall miRNA profiling of this cell line cohort is the identification of a signature of 42 differentially expressed miRNAs, which discriminates between basal-like and normal-like/claudin-low breast cancer cell lines. These miRNAs together with the ER-associated miRNAs are the major determinants of the overall clustering of the cell lines. Importantly, this signature includes all four members of the *hsa-miR-200 *family (*hsa-miR-200a, hsa-miR-200b, hsa-miR-200c, hsa-miR-141*), *hsa-miR-155*, and *hsa-miR-622 *miRNAs. Several studies have implicated these miRNAs to be involved in epithelial-mesenchymal transition (EMT), to be related to the stem-cell-like phenotype, and to be associated with a switch in paclitaxel responsiveness [44-47]. In breast cancer, these miRNAs are known to regulate the EMT process by targeting the ZEB family of the transcription factors through an active negative feedback loop [48,49]. Importantly, the ZEB family of transcription factors was reported to be a repressor of E-cadherin expression in several epithelial carcinomas including breast carcinoma.

Interestingly, we observed a significant inverse correlation between the expression levels of all miRNAs of the *hsa-miR-200 *family and the mRNA expression level of the ZEB1 transcription factor, which is a well-known target of this miRNA family (see Table S20 in Additional file 1). Additionally, our finding that the *hsa-miR-200 *cluster showed lower expression in normal-like/claudin-low breast cancer cell lines fits with the fundamental literature that these miRNAs are indeed involved in EMT, because the majority of the normal-like/claudin-low cell lines lack E-cadherin protein expression, exhibit low claudin expression, and generally display EMT-like features as well as the breast cancer stem cell phenotype (CD44^high^/CD24^low^) [50-53]. Other known significant miRNAs of this signature are *hsa-miR-155 *and *hsa-miR-622*, which were also linked to enhanced tumorigenesis in various cancer types besides breast cancer [54,55]. In concordance with this, we recently found that the normal-like/claudin-low cell lines with low expression of these EMT-related miRNAs show highly aggressive growth characteristics *in vivo *when raised as xenografts in nude mice (M Riaz and colleagues, unpublished data). Importantly, this signature includes three previously unknown miRNAs (*hsa-miR-492, hsa-miR-26b, hsa-miR-617*; fold change ≥ 1.5) found to be associated with cell lines frequently showing EMT-like characteristics.

We observed that the miRNA signatures associated with *E-cadherin *mutation and promoter hypermethylation include distinct miRNAs. This may point to an existence of unique biology at the miRNA level in tumors that show *E-cadherin *inactivation due to gene mutation rather than those that lose *E-cadherin *expression due to promoter hypermethylation. Important to note is that *E-cadherin *promoter hypermethylated cell lines include all normal-like/claudin-low cell lines, and most EMT-related miRNAs were also associated with *E-cadherin *promoter hypermethylation.

With respect to common genetic aberrations present in breast cancer, our study reveals differentially expressed miRNA signatures associated with commonly mutated tumor suppressors and oncogenes. Most significantly, a signature of 30 miRNAs associated with *p16^INK4a ^*mutant cell lines can strongly discriminate between mutant and wild-type ER-negative cell lines. *P16^INK4a ^*is a tumor suppressor gene located on chromosomal band 9p21 that has been frequently altered in many human cancers [56]. *P16^INK4a ^*regulates cell cycle progression by targeting CDK4/6 through the pRb signaling pathway. Interestingly, this signature includes some miRNAs that are involved in cell cycle regulation. For instance, *hsa-miR-100*, which is highly expressed in the *p16^INK4a ^*wild-type cell lines, targets the *RBSP3 *gene that in acute myeloid leukemia regulates the cell cycle through partial modulation of pRB/E2F1 [57]. *hsa-miR-34a *has been reported as a suppressor of cell proliferation and migration in colon cancer [58] and its mechanism of growth inhibition also involves cell cycle arrest followed by apoptosis [59]. Besides this, we found a few miRNAs to be only associated with mutation in *BRCA1, TP53 *and *PIK3CA/PTEN*; these observed associations require further independent validation in breast cancer specimens.

Our finding that the differential expression of miRNAs is associated with ERBB2-overexpressing luminal-type cell lines is also intriguing since the miRNAs showing elevated expression in ERBB2-positive cell lines (fold change ≥ 1.5) are not located on the ERBB2 amplicon (17q12) and thus may be regulated indirectly by genes in the ERBB2 amplicon. It is also important to mention that a majority of these miRNAs have already been implicated in various types of epithelial carcinoma including breast cancer [60-64]. One should, however, note that our study is associative and does not necessarily reveal causality. We therefore propose functional studies on these miRNAs to reveal more biological insights into their role regarding ERBB2 overexpression in breast cancer.

Finally, we also identified 12 miRNAs to be associated with CNVs in breast cancer cell lines (see Table S17 in Additional file 1). The majority of these miRNAs (*hsa-miR-130a, hsa-miR-93, hsa-miR-383, hsa-miR29c, hsa-miR-382, hsa-miR-31*) were already found to be located in regions that exhibited DNA copy number abnormalities in breast cancer tumors [65]. Importantly, this repertoire of miRNAs includes *hsa-miR-22 *previously shown to be regulated by ER [66] and we provide evidence that it can also be regulated by the loss of the locus containing this miRNA. Six miRNAs of this repertoire are located in the genomic regions containing known protein coding genes. All miRNAs of this repertoire have also been implicated in various cancers but a thorough validation of these miRNAs with respect to DNA copy number changes in clinical specimen is imperative.

## Conclusion

In summary, our analyses show that the dichotomy in breast cancer cell lines is, in line with the general consensus in the field, related to ER status. However, part of this dichotomy may be related to ER-independent luminal versus basal differentiation status of cell lines. Secondly, another prominent dichotomy observed among breast cancer cell lines discriminating basal-like cell lines from normal-like/claudin-low cell lines involves miRNAs related to EMT and/or stemness. Furthermore, we reveal sets of miRNAs associated with genes frequently amplified (ERBB2) or mutated (*p16^INK4a^, PIK3CA *and/or *PTEN, E-cadherin, BRCA1*) in breast cancer cell lines and thus these miRNAs may contribute to the function of these oncogenes/tumor suppressors. Finally, certain miRNAs themselves are located in genomic regions, which frequently show genetic aberrations, implying that these miRNAs themselves may have a potential driver role or contribute to known drivers in these genomic regions. Our current findings call for further validation of these signatures in clinical specimens. Importantly, our study provides a unique molecular miRNA portrait of human breast cancer cell lines, which can be exploited for functional studies of clinically important miRNAs.

## Abbreviations

BRCA1: breast cancer 1; BST2: bone marrow stromal cell antigen 2; CDK: cyclin-dependent kinase; CK: cytokeratin; CNV: copy number variation; EMT: epithelial-mesenchymal transition; ER: estrogen receptor; ERBB2: erythroblastic leukemia viral oncogene homolog 2; ESR1: estrogen receptor; FABP7: fatty acid binding protein 7; FOXA1: forkhead box protein A1; GRB7: growth factor receptor-bound protein 7; JNK: c-Jun N-terminal kinase; KRT: keratin; miRNA: microRNA; PI3K: phosphoinositide 3-kinase; PCR: polymerase chain reaction; PTEN: phosphatase and tensin homolog; RB: retinoblastoma protein; SNP: single nucleotide polymorphism; STR: short tandem repeat; TFF3: trefoil factor 3; TP53: tumor protein 53; UTR: untranslated region.

## Competing interests

The authors declare that they have no competing interests.

## Authors' contributions

JAF, JWMM, EAW, and MR designed the study. MR, JAF and JWMM wrote the manuscript. MTMvJ and AWMB performed the miRNA expression analysis. JL and AH performed gene mutation analysis. JH performed STR analysis. BO and WJCP-vdS carried out RNA isolation and quantification. AAJH, MS, and MR performed the statistical data analyses. All authors approved the final version of the manuscript.

## Supplementary Material

Additional file 1 **an Excel file of Tables S1 to S20 containing lists of differentially expressed miRNAs associated with breast cancer molecular subtypes and recurrent genetic aberrations**. Click here for file

Additional file 2 **Figure S1 showing differential expression of miRNAs between ER-positive and ER-negative human breast cancer cell lines**. Cell lines have been grouped into ER-positive and ER-negative groups based on their mRNA expression levels measured by microarray. Yellow and blue, high and low overall similarity of samples in miRNA expression, respectively. Figure S2 showing differential expression of miRNAs between cell lines mutant and wild-type for *BRCA1 *and *PIK3CA/PTEN *genes. (A) miRNA differential expression associated with *PIK3CA/PTEN *mutations in all, the luminal-group, and the ER-negative/basal-group of breast cancer cell lines. (B) miRNA differential expression associated with *BRCA1 *mutation. Yellow and blue, high and low overall similarity of samples in miRNA expression, respectively. Figure S3 showing differential expression miRNAs between cell lines mutant and wild-type for the *TP53 *gene. Cell lines with *TP53 *heterozygous mutation (OCUB-F) and with undetermined *TP53 *mutation status (HCC202, HCC1395, HCC1500) were excluded from the analysis. Yellow and blue, high and low overall similarity of samples in miRNA expression, respectively. Figure S4 showing comparison of median genomic distances of ER binding sites and significantly and nonsignificantly differentially associated miRNAs with ER status of the cell lines. The Mann-Whitney test was used to compare the median genomic distances of ER binding sites and miRNAs. Click here for file
