# Supplementary figures and images for: miRNA expression profiling of 51 human breast cancer cell lines reveals subtype and driver mutation-specific miRNAs
Source: Breast Cancer Res. 2013 Apr 19;15(2):R33. doi: 10.1186/bcr3415 (PMC3672661; doi:10.1186/bcr3415)

Figure S1

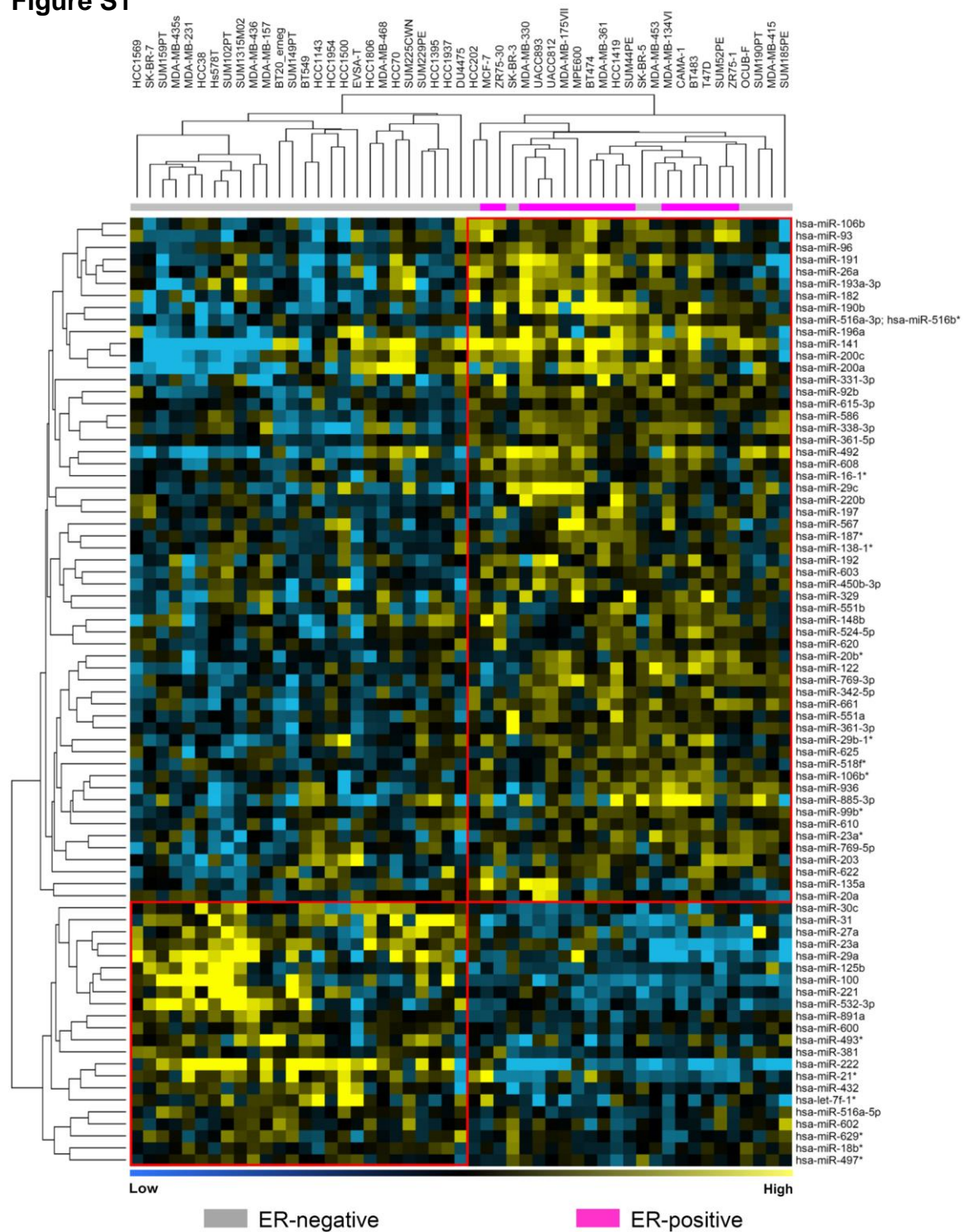

### Figure S2

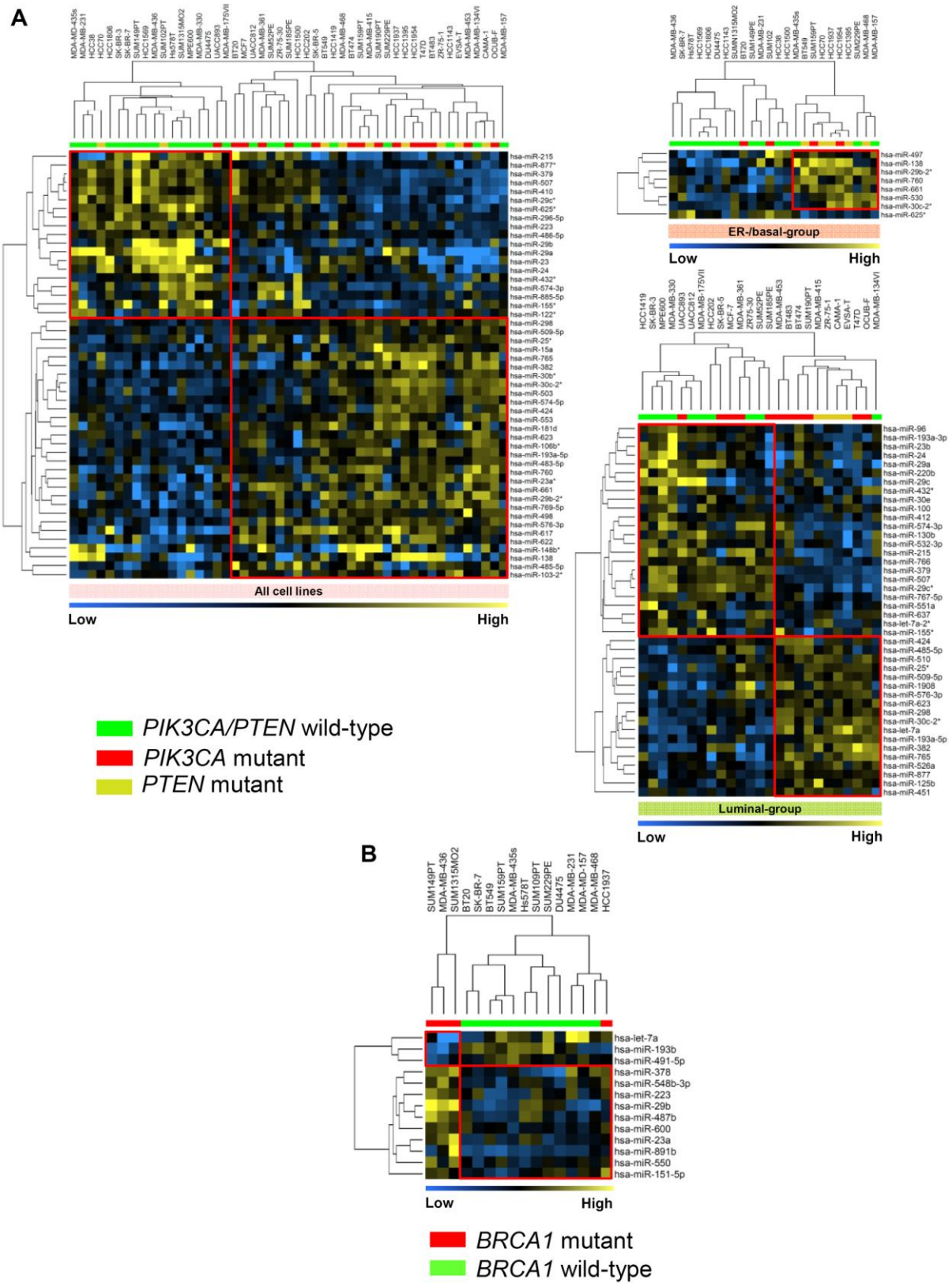

Figure S3

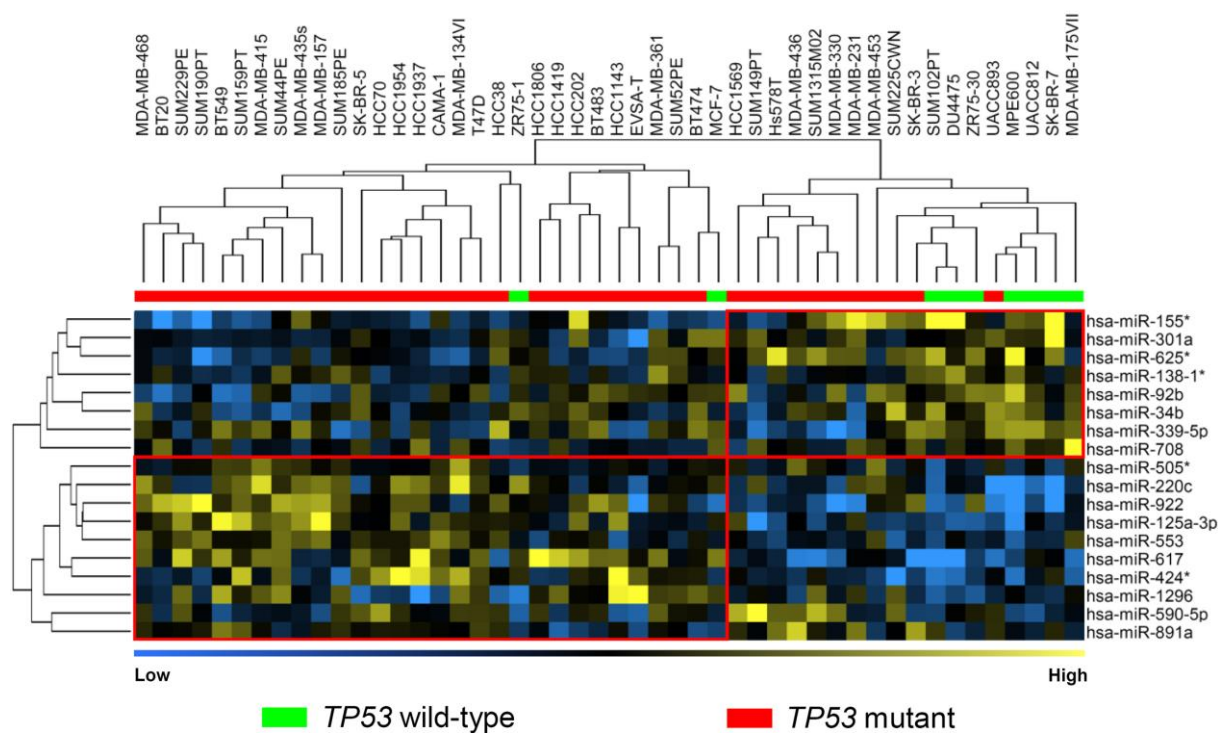

**Figure S4**

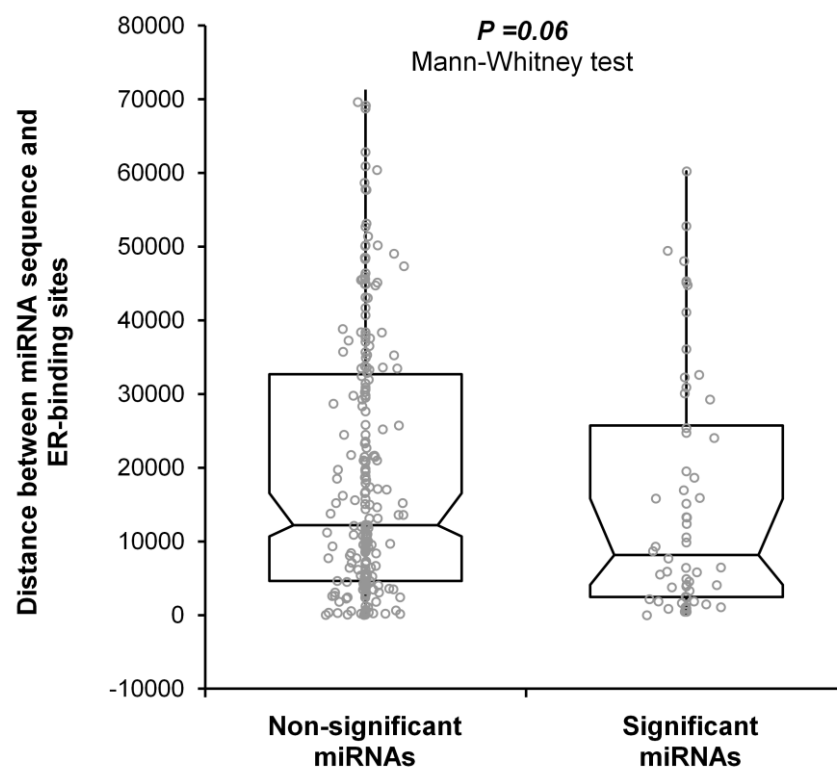

Supplement: Additional file 2 — Figure S1 showing differential expression of miRNAs between ER-positive and ER-negative human breast cancer cell lines. Cell lines have been grouped into ER-positive and ER-negative groups based on their mRNA expression levels measured by microarray. Yellow and blue, high and low overall similarity of samples in miRNA expression, respectively. Figure S2 showing differential expression of miRNAs between cell lines mutant and wild-type for BRCA1 and PIK3CA/PTEN genes. (A) miRNA differential expression associated with PIK3CA/PTEN mutations in all, the luminal-group, and the ER-negative/basal-group of breast cancer cell lines. (B) miRNA differential expression associated with BRCA1 mutation. Yellow and blue, high and low overall similarity of samples in miRNA expression, respectively. Figure S3 showing differential expression miRNAs between cell lines mutant and wild-type for the TP53 gene. Cell lines with TP53 heterozygous mutation (OCUB-F) and with undetermined TP53 mutation status (HCC202, HCC1395, HCC1500) were excluded from the analysis. Yellow and blue, high and low overall similarity of samples in miRNA expression, respectively. Figure S4 showing comparison of median genomic distances of ER binding sites and significantly and nonsignificantly differentially associated miRNAs with ER status of the cell lines. The Mann-Whitney test was used to compare the median genomic distances of ER binding sites and miRNAs. [file bcr3415-S2.PDF]
